# Nurse Leadership Post COVID Pandemic—A Framework for Digital Healthcare Innovation and Transformation

**DOI:** 10.1177/23779608231160465

**Published:** 2023-03-02

**Authors:** Monica Fletcher, Carol Read, Luciana D-Adderio

**Affiliations:** 1 172239 The University of Edinburgh Usher Institute of Population Health Sciences and Informatics, Edinburgh, UK

**Keywords:** digital+, framework, nursing, practice, innovation

## Abstract

**Background:**

The COVID-19 pandemic generated a series of profound and unprecedented challenges for health and social care systems and those frontline clinicians responsible for delivering services including nurses. One consequence has been the rapid and widespread introduction of a range of digital tools, solutions, and initiatives. In the United Kingdom, this has required clinical leadership to drive implementation and adoption of digital innovations across the system, ranging from those in senior executive board level positions to those on the frontline.

**Findings:**

This commentary presents a framework highlighting the breadth of digital transformations which emerged as a consequence of the U.K. health and social care systems’ response to the COVID-19 crisis. The framework outlines the different levels of digital transformation, ranging from what we have termed “ceremonial adoption” to isolated automation, organizational integration, and full systems integration. We reflect on the nursing leadership practices that need to be in place to support these changes.

**Conclusion:**

Whilst acknowledging the extraordinary results achieved by the COVID-19 driven tsunami of digital transformation, we reflect on the essential steps required to translate these nascent, isolated efforts into fully integrated, long-term solutions. We also offer recommendations for clinical digital leaders and suggest steps that will be crucial to translate the temporary and/or limited interventions into effective, permanent features of our health and social care systems, while also providing a platform on which to build future digital capabilities. We will inevitably continue to see an increase in the use of technology in everyday clinical practice, and nurses are well positioned to take a lead in its widespread adoption.

## Introduction

The U.K. National Health Service (NHS) has traditionally demonstrated inertia towards digital innovation and change. A recent report registered the consensus that digital transformation in the U.K. healthcare sector was “slow, expensive, and challenging” (Taylor & Ferris, 2020). Despite efforts to introduce new policies, including the January 2019 NHS Long Term Plan, which was intended to drive wider adoption of digital technologies, the report identified “a growing digital divide between policy ambition and the reality on the front line.” Obstacles included substantial variation in digital maturity of different hospitals, staff, and patients’ confidence in, and willingness to use technologies, the funding available to invest in the IT infrastructure, and the skills and talent needed to implement the much-needed digital interventions. This resulted in digital transformation being confined to isolated pockets within the NHS.

The unanticipated COVID-19 crisis overcame many of these barriers, radically accelerating the adoption of digital tools and technologies within the space of just a few months. The need to urgently triage and treat large numbers of patients with acute respiratory illness, protect the healthcare workforce and ensure they were able to treat the sick, and shield the elderly and most vulnerable from becoming infected, has resulted in the fast adoption of a number of innovations (Robbins et al., 2020). Nurses among others were rapidly mobilized to adopt new patient management systems, ranging from the widespread use of telemedicine consultation approaches for both primary and secondary care, to more complex video conference-based telemedicine or app-based solutions (Hollander & Carr, 2020). As a consequence of the COVID crisis, substantial and pervasive changes have been observed in a number of key areas. These include technology, for example, telephone and online triage (Castle-Clark & Imison, 2016), use of teleconferencing for e-consultations (Greenhalgh et al., 2016), electronic prescriptions (Franklin et al., 2014), and tracking and tracing applications (Dowey, 2020); people (e.g., remote working for some staff and admin personnel), processes and practices (e.g., virtual clinics and appointments, virtual care home ward rounds by video, rapid data-sharing agreements); and organizational and professional culture and structure (e.g., integrated care teams). This has led a range of health sector experts to suggest that “the healthcare of tomorrow will look completely different from today” (Taylor & Ferris, 2020). How could this sudden, radical change take place against all predictions and in the context of such substantial and deeply entrenched cultural, infrastructural, and organizational obstacles?

Our insights are based on a combination of our own opinions and those of a range of expert digitally orientated clinicians including nurses, who are all alumni of the NHS Digital Academy Leadership Programme (Inglis-Humphrey et al., 2022). We believe this gets closer to the realities of COVID and post-COVID U.K. healthcare digital innovation and transformation by highlighting some of the less apparent dynamics.

## Healthcare Digital Transformation After COVID-19

Our first observation is that not all the changes introduced have been material. We have found evidence of superficial changes prompted by the need to align with new digitally oriented care guidelines, whilst also keeping up with the rapidly escalating expectations of the healthcare sector by government, press, and the public. Some of the resources used to implement digital change, for example, were existing resources moved from other areas of the healthcare system to frontline CV-19 activities. The issue of resourcing raises concerns as to whether the progress achieved may be sustainable. The amount of debt accumulated during the fight against CV-19 and the current and projected state of the U.K. economy also raises doubts over the resources that might be directed towards further digital innovation initiatives in the years to come.

Secondly, the reality of the extent of change is not in keeping with that has been suggested (Higgs, 2020). The current data shows an extraordinary rate of uptake of some digital technologies such as teleconferencing used for facilitating online consultations with Practice Nurses, GPs, and hospital healthcare providers. However, these statistics are often not comparing change against the same population because of delayed presentation, as substantial numbers of service users preferred to postpone their needs rather than taking the risk of catching infection by spending time being treated in a hospital or care facility.

Another issue is the uneven distribution of users across different parts of the U.K. healthcare system, due to the limited access to technology and/or lack of basic digital skills. The lack of uniformity also concerns the variable fit of digital tools and interventions across different clinical practices (some practices lending themselves more readily to digital transformation), as well as different health conditions—some being less suitable for remote consultation (Greenhalgh et al., 2020).

More significant is the observation that meaningful digital transformation must go well beyond the straightforward substitution of new technologies and tools for old ones, requiring instead the reconfiguration of how health care is delivered and current practices. Even the most innovative digital intervention will not afford any real advantages unless it is integrated into the flow of work. Looking more closely into the use of e-prescribing during the COVID crisis, for example, our clinical experts observed that some of the perceived change has simply been a matter of performing the practice as before, but with some minor adaptations which, in some cases, have produced complex workarounds and increased paperwork. This is a typical outcome of trying to introduce new tools without also changing practices: if the process is sub-optimal before being automated, it will remain sub-optimal and an opportunity for process improvement will have been missed. A related example is electronic referral, whereby the use of NHS email with word documents appears to have simply replaced manual form filling, faxing or posting. Such cases, only the medium will have changed, not the burden on the clinician, or on the system. To benefit from this, or similar kinds of interventions, digital tools, resources, practices, and capabilities would have to be integrated within NHS sites as well as across the wider healthcare system, to achieve the synergies required for the innovation to realize its potential benefits.

## Current Insights and Interpretations

Drawing on the different ways in which our expert clinicians described how changes were implemented as part of the response to the CV-19 crisis, we were able to identify four kinds, or levels, of adoption (Table 1). The first is what we name partial or “**ceremonial adoption,”** involving either superficial (non-transformative) change, or no real change at all (Greenhalgh et al., 2017). Second, is what we call “**islands of automation,”** namely cases in which some digital change had occurred, but which was limited to the implementation of minor, isolated changes or simple tools with limited capabilities and/or connections to the rest of the system. Third, **organizational change** in the form of interventions which involved the reconfiguration of healthcare practices and workflows to fit the tool, and vice-versa. And fourth, **systemic change**, that is change initiatives which were effectively integrated with expertise and resources planning across healthcare sites and the various levels of the healthcare system, change primarily oriented towards addressing actual patient/user needs. Our initial investigation suggests that the digital transformation driven by the CV-19 crisis has been mostly of the first and second kind, with less evidence of the third, and very limited evidence of the fourth. If this initial finding was replicated through more extensive studies, it would bear important implications for digital transformation leaders—during times of crisis, and much further beyond.

**Table 1.** A Framework for Digital Innovation.

Types of digital adoption	Description	Example: video consultations/telemedicine
*non-adoption or ceremonial adoption*	No uptake (tech./tool is rejected or disused) or symbolic conformity (tech/tool is adopted only in discourse, not in practice)	Video consultation/telemedicine implemented but not used
*islands of automation*	Isolated adoption (tech/tool as isolated “solution” not connected to organizational processes/structures)	Video consultation/telemedicine adopted as an isolated solution enables basic remote consultation capabilities
*organizational/workflow integration*	Tech/tool is integrated into organizational practices and routines, reconfiguring them	Video consultation/telemedicine integrated into workflows/practices allows aligning admin practices to support virtual appointments
*systemic change*	Tech/tool, knowledge, cultures, infrastructures and resources integrated through healthcare system; focus on problem/patient-centric; automation	Video consultation/telemedicine allows clinicians & patients to view/exchange data (via integration with the Electronic Health Record)

## Implications for Digital Nurse Leaders

What does taking a systemic view of digital innovation and transformation mean for healthcare leaders?

It is important to abandon a reactive and fragmented approach to digital transformation in exchange for an integrated problem- and patient-centric approach. This involves starting with the users, involving them in the conversation and identifying their needs; turning those needs into a problem that can be solved through the introduction of a digital initiative by directing resources and training towards achieving a set of desirable outcomes; and finally understanding how existing processes, practices, and workflows need to be reconfigured to deliver those outcomes. It is important to consider the depth of nursing knowledge that can be applied to provide holistic care alongside the medical model.

The knowledge and experience acquired through (both successful and failed) digital initiatives implementation needs to be captured at all levels of the healthcare system and integrated into policy and practical learning which can guide future interventions. Nurses and other clinicians on the frontline are crucial in this aspect as they can support the translation of learning acquired on the ground into higher-level policy. This calls for distributive leadership across the healthcare system so that leaders at all levels are actively engaged in—and share responsibility for—the digital innovation and transformation strategy objectives.

The NHS is a complex adaptive system and an acceptance that digital innovation and transformation in the healthcare system differs from other sectors is required. In healthcare, processes, systems, incentives, and drivers are substantially different from other sectors such as financial services, and they are often not aligned. Part of the problem is the focus on measuring process rather than outcomes (e.g., patient utility or welfare). Measuring outcomes across the system, enabled by a higher level of knowledge and process integration, could help align the dispersed incentives, drive transformation, and bring in better products and services that improve utility to the user/patient. The use of a socio-technical model such as that described by Sittig and Singh (2015) could facilitate a deeper understanding of the adoption of digital technologies within the NHS.

Finally, it is important that clinical nurse leaders are educated to acquire a system-level understanding of digital transformation. Digital transformation must start in the minds of the leaders. They need a deeper understanding of how new digital tools and technologies can—and indeed should—be integrated across healthcare processes, practices, organizations, and the overall system in a way to create an effective transformation capability rather than one-off, time-limited, local interventions. To avoid narrow approaches, training should also involve learning from past initiatives such as Connecting for Health (Khong et al., 2008), and Global Digital Exemplars (NHS England 2017), and multi-professional colleagues in the United Kingdom and beyond.

To future proof digital transformation more comprehensively, both clinical nurse leaders and nursing educationalists need to ensure that digital competencies embedded in practice and educational pathways are available from novice to expert nurse roles. These include pre-registration programs through to specific post-graduate Digital Leadership programs such as provided by the NHS Digital Academy in the United Kingdom. Davis et al. (2022) highlight ways to achieve this at pace through blended learning and communities of practice, which help to use both experience and adoption of new technologies as they become relevant to healthcare.

Whilst this paper is in press The Phillips Ives Nursing and Midwifery Review is taking place. This year's long review due to report in Summer 2023 builds on the findings and recommendations from The Topol Review (2019) and aims to determine the needs of the nursing and midwifery workforce to deliver health care in the digital age over the next 5 to 20 years. A comprehensive review is taking place across seven themes:

Specialist practice (Professionalism)
Data capture and use in nursing and midwifery practice
Population health
Regulation and education standards
Place-based person-centered care supported by tech
Geonomics in nursing and midwifery practice
AI in nursing and midwifery

This multi-strand approach seeks to capture both National and International expert opinion and thought leadership, case studies, benchmarking, crowdsourcing ideas from nurses and midwifes in practice and the opportunity to participate in the consultation process. The findings from the review will provide research-based and critically appraised recommendations to ensure the breadth of ambition and scope of future practice of pre- and post-nursing is captured.

## Conclusions

Recent publications (Greenhalgh et al., 2017; The Topol Review, 2019) insightfully describe digital change as being only partially about the innovation itself but mostly about organizational and system behaviors. Our findings support this statement while adding a conceptual framework which identifies four different kinds of digital innovation, culminating in a systemic approach revolving around establishing a multi-level, integrated learning capability centrally focused on patient/user needs. Within this context, nurse-led leadership and innovation plays a fundamental role for realizing a digitally enabled care environment that makes the best use of technological advances. This paper seeks to contribute awareness of both the challenge and the exciting opportunities for nurses at all levels to be part of the digital transformation of health and social care and contribute to the ongoing debate on the nature of nursing in the future.
